# Intraoperative Ultrasound During Surgical Exploration in Patients with Pancreatic Cancer and Vascular Involvement (ULTRAPANC): A Prospective Multicenter Study

**DOI:** 10.1245/s10434-023-13112-3

**Published:** 2023-02-11

**Authors:** Nynke Michiels, Deesje Doppenberg, Jesse V. Groen, Eran van Veldhuisen, Bert A. Bonsing, Olivier R. Busch, A. Stijn L. P. Crobach, Otto M. van Delden, Susan van Dieren, Arantza Farina, Ignace H. J. T. de Hingh, Rob Hurks, Joost Nederend, Shirin Shahbazi Feshtali, Yeliz Tank, A. L. Vahrmeijer, Martin Wasser, Marc G. Besselink, J. Sven D. Mieog, for the Dutch Pancreatic Cancer Group

**Affiliations:** 1 Department of Surgery, Leiden University Medical Center, Leiden, The Netherlands; 2 Department of Surgery, Amsterdam UMC, University of Amsterdam, Amsterdam, The Netherlands; 3 Cancer Center Amsterdam, Amsterdam, The Netherlands; 4 Department of Pathology, Leiden University Medical Center, Leiden, The Netherlands; 5 Department of Interventional Radiology, Amsterdam UMC, University of Amsterdam, Amsterdam, The Netherlands; 6 Department of Pathology, Amsterdam UMC, University of Amsterdam, Amsterdam, The Netherlands; 7 Department of Surgery, Catharina Hospital, Eindhoven, The Netherlands; 8 Department of Radiology, Catharina Hospital, Eindhoven, The Netherlands; 9 Department of Radiology, Leiden University Medical Center, Leiden, The Netherlands

## Abstract

**Background:**

Determining the resectability of pancreatic cancer with vascular involvement on preoperative computed tomography imaging remains challenging, especially following preoperative chemotherapy and chemoradiotherapy. Intraoperative ultrasound (IOUS) may provide real-time additional information, but prospective multicenter series confirming its value are lacking.

**Patients and Methods:**

This prospective multicenter study included patients undergoing surgical exploration for pancreatic cancer with vascular involvement. All patients underwent IOUS at the start of explorative laparotomy. Primary outcomes were resectability status as defined by the National Comprehensive Cancer Network and the extent of vascular involvement.

**Results:**

Overall, 85 patients were included, of whom 74 (87%) were post preoperative chemotherapy, and mostly following FOLFIRINOX regimen (*n* = 57; 76%). On the basis of preoperative imaging, 34 (40%) patients were staged as resectable (RPC), 32 (38%) borderline resectable (BRPC), and 19 (22%) locally advanced pancreatic cancer (LAPC). IOUS changed the resectability status in 32/85 (38%) patients (*p* < 0.001), including 8/19 (42%) patients with LAPC who were downstaged (4 to BRPC, 4 to RPC), and 22/32 (69%) patients with BRPC who were downstaged to RPC. Among patients with presumed superior mesenteric artery (SMA) involvement, 20/28 (71%) had no SMA involvement on IOUS. In 15 of these 20 patients a pancreatic resection was performed, all with R0 SMA margin.

**Conclusion:**

IOUS during surgical exploration for pancreatic cancer and vascular involvement downstaged the resectability status in over one-third of patients, which could facilitate progress during surgical exploration. This finding should be confirmed by larger studies, including detailed pathology assessment.

*Trial Registration* www.trialregister.nl (NL7621).

**Supplementary Information:**

The online version contains supplementary material available at 10.1245/s10434-023-13112-3.

Pancreatic ductal adenocarcinoma (hereafter: pancreatic cancer) is notorious for its high cancer-related mortality. Radical surgical resection (R0 resection), in combination with systemic treatment, provides the best chance for long-term survival.^1–3^ Among patients with non-metastasized pancreatic cancer, 50–60% are not eligible for upfront surgical resection, as they present with borderline resectable pancreatic cancer (BRPC) or locally advanced pancreatic cancer (LAPC).^4,5^ These patients are generally offered chemotherapy or chemoradiotherapy as induction therapy to increase the feasibility of a curative resection.^6^

Accurate assessment of resectability status on preoperative computed tomography (CT) or magnetic resonance imaging (MRI) and during surgical exploration remains troublesome, especially following preoperative therapy.^7,8^ Preoperative therapy often causes inflammation, fibrosis, or both, which is difficult to distinguish from vital tumor tissue. This may lead to overestimation of tumor extension and vascular involvement.^9–11^ During surgical exploration, surgeons have to judge vascular ingrowth by distinguishing vital tumor tissue from fibrosis and normal tissue, which is quite challenging and may require lengthy explorations with numerous frozen sections. In addition to preoperative imaging, intraoperative ultrasound (IOUS) is a relatively easy approach to potentially improve the assessment of vascular involvement, because of its better spatial resolution compared with CT imaging. Thereby, it may improve staging and guide resection.^12,13^

Our group previously published two smaller studies suggesting that IOUS can be useful during surgical exploration of pancreatic cancer, but larger studies were recommended.^14,15^ The current prospective multicenter study investigated the impact of IOUS in assessing resectability status of patients with pancreatic cancer, with focus on the assessment of vascular involvement.

## Patients and Methods

### Study Design and Patient Selection

The ULTRAPANC study is a prospective, nonrandomized, multicenter cohort study including patients from three centers of the Dutch Pancreatic Cancer Group (DPCG), between October 2018 and April 2021. Inclusion criteria were patients aged 18 years and older with (suspected) pancreatic cancer, vascular involvement on preoperative CT and/or MRI imaging, regardless of tumor location, with or without preoperative therapy and plans for curative intended surgery. Exclusion criteria were periampullary cancer of nonpancreatic origin and pancreatic cancer in the absence of vascular involvement on preoperative imaging. Informed consent was obtained for the prospective collection of clinical data. The study was approved by the Medical Ethics Committee of the Leiden University Medical Center. All data were collected in an online secured database (Castor EDC, CIWIT B.V., Amsterdam, The Netherlands). This study is reported according to the Strengthening the Reporting of Observational Studies in Epidemiology (STROBE) guidelines.^16^

### Outcomes

Primary outcomes were the resectability status according to the National Comprehensive Cancer Network (NCCN) criteria and the presence, and extent, of vascular involvement. Resectability status was defined according to the NCCN guidelines.^5^ Herein, resectable pancreatic cancer (RPC) is defined as no arterial [specifically superior mesenteric artery (SMA) and/or common hepatic artery and/or celiac axis (CA)] contact, and ≤ 180° venous contact (specifically portal vein (PV) and/or superior mesenteric vein (SMV)); BRPC is defined as ≤ 180° arterial contact and/or reconstructible venous contact; LAPC is defined as > 180° arterial involvement and/or unreconstructible venous involvement.

Subanalyses regarding resectability status were performed using the criteria of the DPCG.^17^ Secondary outcome was the resection margin status upon pathological examination. Radical resection (R0) was defined as no microscopic residual disease, R1 as microscopic residual disease within 1 mm of the resection margin, and R2 as macroscopic residual disease.^18^

### Imaging Modalities

Preoperatively, tumor extension was determined on contrast-enhanced abdominal CT or MRI (or both), which were acquired according to local protocols. Generally, imaging consisted of at least a pancreatic phase (i.e., late arterial) and a portal venous phase with slices of maximum 2 mm, according to the Dutch guidelines.^19,20^ Axial, coronal, and sagittal reconstructions of the most recent preoperative imaging were used for evaluation. Evaluation was performed by experienced abdominal radiologists and discussed afterwards with pancreatic surgeons during multidisciplinary pancreatic team meetings. Based on this final evaluation, a Case Report Form (CRF; Supplementary Fig. A1) was filled out. During surgical exploration, all patients underwent IOUS by experienced abdominal or interventional radiologists.^13,21^ The findings of IOUS were directly communicated to the surgical team, and subsequently all patients were surgically explored as preoperatively planned. By design, radiologists were not blinded for preoperative imaging results. A standardized scoring form was used to report the findings of preoperative imaging and IOUS (Supplementary Fig. A1).

### Statistical Analyses

We expected that IOUS would change the resectability status in at least 15% of patients.^14,15^ To detect an odds ratio of 7.00, a sample size of 85 patients was calculated using a two-sided McNemar power test with a significance level of 0.05 and a power of 80%. Subgroup analyses were performed comparing patients with and without preoperative therapy. Statistical analyses were performed using IBM SPSS Statistics version 25.0 (IBM, USA). Continuous variables are presented as mean [standard deviation (SD)] or median [interquartile range (IQR)] and were compared using an unpaired *t*-test or Mann–Whitney *U* test, depending on their distribution. Categorical data are presented as frequencies (percentages) and were compared using the chi-square or Fisher’s exact test. Differences between preoperative imaging and IOUS were analyzed using the McNemar–Bowker test of symmetry. Diagnostic accuracy was determined using sensitivity/specificity analyses. A *p* value < 0.05 was considered as statistically significant.

## Results

### Baseline Characteristics

Overall, 85 patients were included in the study. Preoperative chemotherapy and chemoradiotherapy was administered to 74 (87%) patients, of which 57 received FOLFIRINOX (median 4 cycles; IQR 4–8) and 17 patients gemcitabine-based therapy (median 3 cycles; IQR 3–3; Table 1). Twelve of these patients underwent additional radiotherapy. The vast majority of patients (76/85) were preoperatively staged by CT imaging, eight by CT and MRI combined and one by MRI only. On preoperative imaging, the mean tumor diameter was 31.9 mm (SD 13.2). Seventy-two (85%) patients showed PV/SMV contact, 28 (33%) SMA contact, 16 (19%) contact with the celiac axis, 18 (21%) with the common hepatic artery, and 21 (25%) with other vessels. On the basis of NCCN resectability criteria, 34 patients (40%) were staged as RPC, 32 (38%) as BRPC, and 19 (22%) as LAPC.

**Table 1:** Patient, treatment, and radiological characteristics

		*N*	%
Total		85	
Age (years)	Mean (SD)	64 (9.6)	
Sex	Male	47	55
	Female	38	45
BMI (kg/m^2^)	Mean (SD)	24.4 (3.5)	
ASA score	I–II	63	55
	III–IV	22	26
Preoperative biliary drainage	No	27	32
	Yes	58	68
Preoperative chemotherapy	No	11	13
	Gemcitabine-based	17	20
	FOLFIRINOX	57	67
Preoperative radiotherapy	No	70	82
	Yes	15	18
Type of preoperative imaging	CT	76	89
	CT + MRI	8	9
	MRI	1	2
Location of tumor^†^	Pancreatic head	70	82
	Distal (left sided) pancreas	14	16
	Unknown	1	2
Radiological response to preoperative therapy*	Complete response	0	0
	Partial response	6	10^#^
	Stable disease	53	88^#^
	Progressive disease	1	2^#^
	Missing	14	
Tumor size on preoperative imaging (mm)	Mean (SD)	31.9 (13.2)	
Preoperative vascular involvement	PV/SMV	72	85
	SMA	28	33
	CA	16	19
	CHA	18	21
	Other	21	25
Preoperative resectability status	Resectable	34	40
	Borderline resectable	32	38
	Locally advanced	19	22
Tumor size on IOUS (mm)	Mean (SD)	29.4 (13.1)	

**†**Pancreatic head tumors include tumors of the pancreatic head, neck, and uncinate process. Distal (left sided) tumors include tumors located in the pancreatic body and tail.

*RECIST criteria

#Percentage of all patients who received preoperative therapy (*n* = 74)

*SD* Standard deviation, *BMI* Body mass index, *ASA* American Society of Anesthesiology, *PV*/*SMV* Portal vein/superior mesenteric vein, *SMA* Superior mesenteric artery, *CA* Celiac axis, *CHA* Common hepatic artery, *IOUS* Intraoperative ultrasound

#### IOUS

Median number of days between preoperative imaging and IOUS was 20 (interquartile range 12.0–28.25). We performed 82 IOUS procedures during open surgery and 3 during minimally invasive surgery. Regarding tumor size, IOUS demonstrated similar results to preoperative imaging (Table 1).

### Resectability Status and Vascular Involvement

**Fig. 1:**
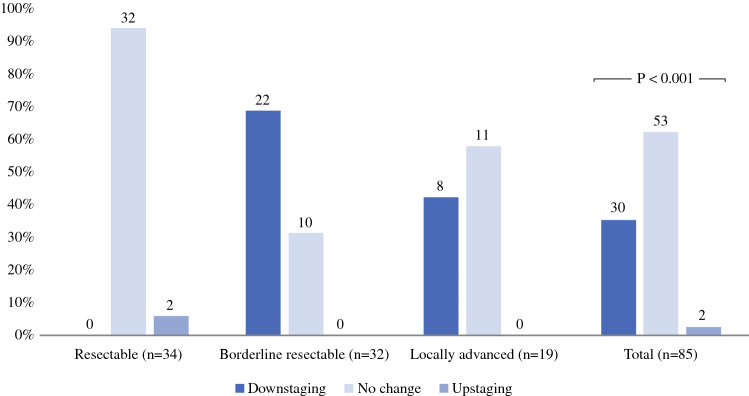
Change in resectability status after IOUS. *IOUS* intraoperative ultrasound

**Fig. 2:**
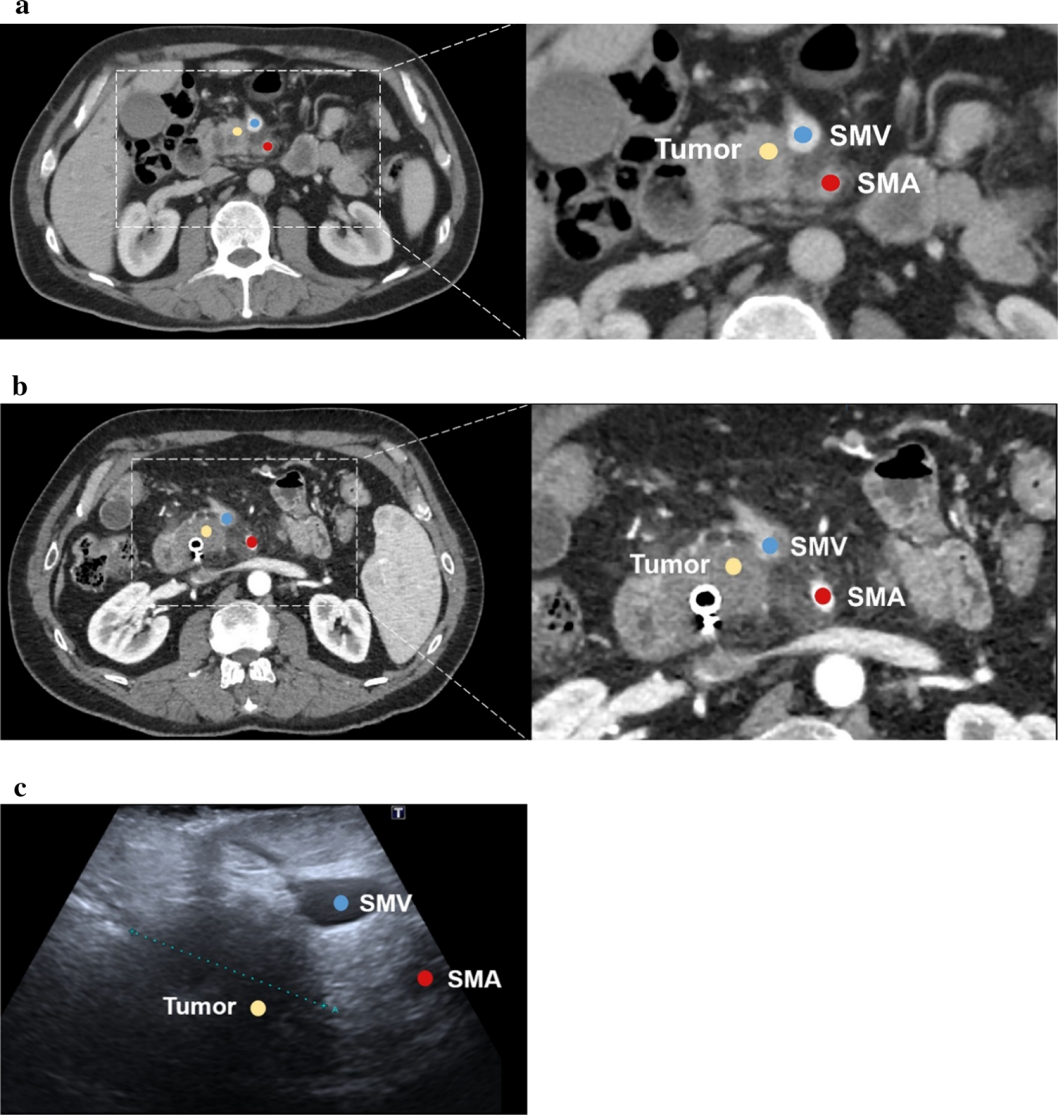
Typical example of downstaging after IOUS. **a** CT imaging at time of diagnosis (before biliary stent placement): involvement of the superior mesenteric artery (SMA; 270–360°) and superior mesenteric vein (SMV; 90–180°). Resectability status at diagnosis: locally advanced (LAPC). **b** CT imaging after completing preoperative chemoradiotherapy: persistent infiltration was seen surrounding the SMA (> 270°) and the superior mesenteric vein (90–180°), essentially unchanged when compared with initial imaging. Staging after preoperative therapy: LAPC. **c** Intraoperative ultrasound (IOUS): at most, 90° contact with the superior mesenteric vein was visible and no involvement of the superior mesenteric artery. Staging after IOUS: resectable. Time between last preoperative imaging and IOUS: 35 days

Overall, evaluation of resectability status changed following IOUS in 32 (38%) patients (*p* < 0.001; Table 2, Fig. 1). Among 32 patients preoperatively staged as BRPC, 22 (69%) were downstaged to resectable disease. Among 19 patients preoperatively staged as LAPC, 8 (42%) were downstaged, of whom 4 were downstaged to BRPC and 4 to resectable disease (Fig. 2). Upstaging was observed in two (6%) patients (from RPC to BRPC), of which both patients had received induction therapy with FOLFIRINOX without additional radiotherapy. In the group of patients treated with preoperative therapy, resectability status changed in 28 (37%) patients (*p* < 0.001), of whom 26/28 were downstaged. In patients without preoperative therapy, resectability status was downstaged in 5/11 (45%), no upstaging was reported. In patients who received chemotherapy in combination with radiotherapy, downstaging was observed in 2/15 and no change in resectability status in 13/15 patients. Change in resectability status based on the criteria of the DPCG is provided in Supplementary Table S1.

**Table 2:** change in resectability status after IOUS

		Resectability status based on IOUS			Total
		Resectable	Borderline resectable	Locally advanced	
Resectability status based on preoperative imaging	Resectable	32 (94%)	**2 (6%)**	**0 (0%)**	34 (100%)
	Borderline resectable	*22 (69%)*	10 (31%)	**0 (0%)**	32 (100%)
	Locally advanced	*4 (21%)*	*4 (21%)*	11 (58%)	19 (100%)
	Total	58 (68%)	16 (19%)	11 (123%)	85 (100%)

The italic cells indicate the patients in which resectability status was downstaged after IOUS. The bold cells indicate the patients in which resectability status was upstaged after IOUS. Overall change in resectability status: 32/85 = 35% (*p* < 0.001)

*IOUS* intraoperative ultrasound

Downstaging was largely attributed to a decrease in arterial involvement during IOUS (SMA, celiac axis, common hepatic artery), rather than venous involvement (PV/SMV; Table 3). If vascular involvement was considered as independent observations, downstaging of preoperative BRPC- or LAPC-categorized arterial involvement was seen in 44/60 (73%), and downstaging of PV/SMV involvement in 5/11 (45%).

**Table 3:** Venous and arterial vascular contact on preoperative imaging versus IOUS

(1) Portal vein/superior mesenteric vein							
		PV/SMV contact on IOUS				Total	*p* value
		No contact	1–180°	> 180°	Unreconstructible		
PV/SMV contact on preoperative imaging	No contact	*10 (77%)*	3 (23%)	0 (0%)	0 (0%)	13 (100%)	0.065
	1–180°	11 (19%)	*45 (76%)*	2 (3)	1 (2)	59 (100%)	
	> 180°	0 (0%)	5 (50%)	*3 (30%)*	2 (20%)	10 (100%)	
	Unreconstructible	0 (0%)	0 (0%)	0 (0%)	*1 (100%)*	1 (100%)	
	Total	21 (25%)	53 (64%)	5 (6%)	4 (5%)	83 (100%)	

(2) Arterial, classified per artery						
		Arterial contact on IOUS			Total	*p* value
		No contact	1–180°	> 180°		
SMA contact on preoperative imaging	No contact	*56 (98%)*	1 (2)	0 (0%)	57 (100%)	< 0.001
	1–180°	17 (77%)	*5 (23%)*	0 (0%)	22 (100%)	
	> 180°	3 (50%)	1 (17%)	*2 (33%)*	6 (100%)	
	Total	76 (90%)	7 (8%)	2 (2%)	85 (100%)	
CA contact on preoperative imaging	No contact	*68 (100%)*	0 (0%)	0 (0%)	68 (100%)	0.004
	1–180°	9 (82%)	*2 (18%)*	0 (0%)	11 (100%)	
	> 180°	2 (67%)	0 (0%)	*1 (33%)*	3 (100%)	
	Total	79 (96%)	2 (3%)	1 (1%)	82 (100%)	
CHA contact on preoperative imaging	No contact	*65 (98%)*	1 (2%)	0 (0%)	66 (100%)	< 0.001
	1–180°	7 (78%)	*2 (22%)*	0 (0%)	9 (100%)	
	> 180°	3 (33%)	2 (22%)	*4 (45%)*	9 (100%)	
	Total	75 (89%)	5 (6%)	4 (5%)	84 (100%)	

Categorization of extent of vascular involvement is based on the resectability criteria. The italic cells indicate the patients in which the amount of vascular involvement as assessed on preoperative imaging was the same as on IOUS

*IOUS* Intraoperative ultrasound, *PV/SMV* Portal vein/superior mesenteric vein, *SMA* Superior mesenteric artery, *CA* Celiac axis, *CHA* Common hepatic artery

### Pancreatic Resection

After exploration and IOUS, 73/85 (88%) patients underwent pancreatic resection. Four patients had metastatic disease and eight had locally unresectable disease, which led to refraining from primary tumor resection. In five of the eight patients with local unresectability, this was seen on both preoperative imaging and IOUS. In two of the eight patients with local unresectability, unresectability was suspected on IOUS and confirmed by frozen sections. In one patient, frozen section revealed cancer surrounding the proper hepatic artery, whereas IOUS did not indicate arterial involvement. In this patient, the radiologist reported difficulties in distinguishing actual tumor tissue from active pancreatitis. In the four patients with metastatic disease, all distant lesions were seen macroscopically and were not an additional finding of IOUS.

In one of the two patients in whom upstaging was observed (both from RPC to BRPC), resection was not performed because of a distant metastasis. In the other patient, a pancreatoduodenectomy without venous resection was performed, although IOUS suggested involvement of over 180°. On pathological examination, this specimen was graded R1 because of a 0.5 mm distance of the tumor to the PV/SMV dissection plane.

Among patients who underwent pancreatic resection, venous resection was performed in 34 (40%) patients. In these patients, venous involvement was indicated by preoperative imaging in 33/34, and by IOUS in 32/34. Vascular resection of the celiac axis was performed in two patients, and of the right hepatic artery in one patient; in each of these patients arterial involvement was indicated by preoperative imaging, as well as by IOUS.

### Pathology

No significant difference was found in mean tumor size upon pathological examination of the resection specimen, compared with IOUS (28.4 mm vs. 29.7 mm). Overall, an R0 resection was obtained in 39 (53%) patients, R1 was reported in 34 (47%) patients. After PV/SMV resection, the PV/SMV margin was R0 in 15/34 (44%) and R1 in 19/34 (56%).

Using R0 status of the vascular resection margin as a reference standard, the specificity after IOUS was higher than that of preoperative imaging alone for estimating PV/SMV (0.34 versus 0.19), and SMA (0.93 versus 0.71) involvement. Of the 28 patients with presumed SMA involvement on preoperative imaging, 20 (71%) had no SMA contact on IOUS (*p* < 0.001). In 15 of these 20 patients, pancreatic resection was subsequently performed and the SMA margin was R0 in all 15 (100%) patients. The remaining five patients did not undergo pancreatic resection. Similarly, IOUS showed no arterial contact, whereas arterial contact was present upon preoperative imaging in 13/16 (81%) patients for celiac axis and 9/18 (50%) for common hepatic artery involvement. Of the 70 patients who showed PV/SMV contact on preoperative imaging, IOUS showed no contact in 11 patients (16%). Nine of these 11 patients underwent pancreatic resection, with 8 (89%) of these showing R0 PV/SMV margin.

## Discussion

This prospective multicenter study found that IOUS, during surgical exploration in patients with pancreatic cancer and suspected vascular involvement, downstaged the resectability status in one-third of the patients. In two-thirds of patients with arterial involvement on preoperative imaging, IOUS demonstrated the absence of arterial contact.

Previous studies on IOUS in pancreatic cancer have mainly focused on the detection of metastases, rather than the evaluation of vascular involvement.^21–24^ A previous prospective monocenter study by our group suggested that IOUS could be useful, as it downstaged the local resectability status in one-third of 38 patients with LAPC after induction chemotherapy.^14^ Another monocenter study by our group included 31 patients with pancreatic ductal adenocarcinoma (PDAC), or periampullary cancer, and indicated more accurate assessment of vascular involvement by IOUS, compared with conventional imaging modalities.^15^ Meanwhile, in the present study, we included a larger sample size of patients with suspected vascular involvement, with or without preoperative therapy, and reported the results for separately venous and arterial involvement and the “combined” overall resectability status. In the present study, 42% of patients with LAPC were downstaged to (borderline) resectable disease. Certainly, downstaging from LAPC to (B) RPC has more clinical significance, as it may allow for a potential curative resection in patients with presumed unresectable disease, whereas downstaging from BRPC to RPC might be less relevant in this setting. However, in these patients, IOUS could improve our certainty regarding which patients to continue the exploration with intent to resection and facilitate the preparations for vascular resection.

Selecting the optimal treatment in patients with LAPC remains challenging, and extensive arterial involvement on preoperative imaging can be a reason to withhold from exploration. This is illustrated by an international survey among 153 surgeons, of which 86% considered themselves as “high-volume” pancreatic surgeons, who reported the reasons for not considering exploration in six case vignettes of patients preoperatively staged as LAPC.^25^ Overall, the main reason not to perform an exploration was presumed arterial involvement on preoperative imaging. On the other hand, a recent study suggested that patients with extensive (>180°) SMA contact and a “halo sign” on preoperative imaging following systemic treatment, may benefit from periadventitial dissection of the SMA to achieve a radical resection.^26^ The multicenter PREOPANC-4 study by the DPCG is currently investigating to what extent implementing the international standards of excellence for LAPC surgery following systemic treatment will increase the resection rate and improve survival in these patients in the Netherlands. The results of the current study, suggesting an overestimation of arterial involvement, in particular SMA involvement, on preoperative imaging, support surgical exploration in patients with LAPC based on arterial contact following systemic treatment. However, considering the limited experience on this topic, this should be investigated further.

Regarding venous contact in PDAC, the International Study Group of Pancreatic Surgery (ISGPS) notes that portomesenteric venous resections should be considered in order to achieve a radical resection.^27^ Despite a proclaimed preference of surgeons for segmental venous resection, a recent Dutch nationwide study showed higher morbidity and postoperative mortality rates after venous segmental resection, compared with wedge resection.^28,29^ Our results suggest a decrease of “clinically relevant” venous involvement, as IOUS showed ≤ 180° PV/SMV involvement in 50% of patients with > 180° PV/VMS involvement on preoperative imaging (Table 3). Considering these results, IOUS has the potential to be a valuable diagnostic modality for accurate detection of a clinically relevant decrease in venous involvement hence optimizing selection of patients requiring venous segmental resection.

Preoperative therapy is increasingly being used in patients with pancreatic cancer and vascular involvement, complicating the assessment of resectability on preoperative imaging. This has been demonstrated by several studies, showing a poor to moderate interobserver agreement^7,30–32^ and discrepancies between radiological and pathological assessment.^32^ In addition, improved surgical techniques lead to more explorations in patients with LAPC. In this light, the need for better diagnostic tools is evident to improve patient selection for potential pancreatic resection. It is hypothesized that the higher resolution of ultrasound, when compared with CT, might improve the distinguishment of vital tumor tissue from peritumoral fibrosis or inflammation, in particular in neurolymphatic tissue, explaining the difference between arterial and venous downstaging, as observed in this study. Transabdominal ultrasound before surgery would be desirable; however, this would be hampered by interference of gas in the stomach and interposition of bowels. The real-time and dynamic imaging in direct contact with the tumor of IOUS possibly facilitates the surgeon during the intraoperative decision-making process and may partially substitute the need for time-consuming frozen sections.

For the interpretation of these outcomes, some limitations should be considered. First, all IOUS procedures were performed by (interventional) radiologists who were not blinded for preoperative imaging results. This was done on purpose to reflect clinical practice, as the added value of IOUS is what matters most. Also, the operating surgeon has detailed knowledge of presumed vascular involvement on preoperative imaging. In other healthcare settings, surgeons may be more interested to perform the IOUS procedures themselves. This could be a topic of future studies. To minimize biased interpretations of the IOUS, a standardized case report form was filled out that categorized intraoperative measures. Second, no validated set of IOUS criteria is available, which may introduce heterogeneity in the observed clinical findings. Third, the correlation with pathology results should be interpreted with caution. Accurate surgical marking of sites suspicious for vascular tumor involvement is often challenging. Therefore, one-to-one pathological examination was not always possible, unless this was explicitly described or marked by the surgeon. Fourth, subanalyses regarding the different preoperative imaging modalities (i.e., comparing IOUS versus CT with IOUS versus MRI) could not be performed. Fifth, this study design predominantly interpreted numeric outcomes, rather than the clinical applicability, such as change in surgical strategy. Therefore, we cannot be sure whether surgical exploration outcomes would have been different without IOUS on the basis of our data, the ultimate outcome (resection or no resection) may have been similar without IOUS. However, it was noticed that IOUS findings strongly guided the exploration process and facilitated targeted frozen sections at remaining areas of potential vascular involvement. Since this was not an endpoint of the current study, these findings were not systematically assessed. However, it does provide a direction for future research objectives. Responding to these limitations, a subsequent study (ULTRAPANC II) is pending, which also assesses the impact of IOUS on clinical decision making and will focus on the correlation between IOUS and pathology results by sampling of discrepant areas between IOUS and preoperative imaging.

In conclusion, this study suggests that the use of IOUS in patients with pancreatic cancer and vascular involvement on preoperative imaging leads to a considerable change in resectability status, mainly caused by the downgrading of arterial involvement. This would be instrumental for surgeons as it provides more confidence in the resectability status and may guide the focus of the surgical exploration to areas where vascular invasion is mostly present. Future studies should investigate whether IOUS has the potential to influence the surgical strategy and increase R0 resection rates in LAPC.

## Supplementary Information

Below is the link to the electronic supplementary material.

Supplementary file1 (DOCX 58 kb)
