# Supplementary material for: Intraoperative Ultrasound During Surgical Exploration in Patients with Pancreatic Cancer and Vascular Involvement (ULTRAPANC): A Prospective Multicenter Study
Source: Ann Surg Oncol. 2023 Feb 11;30(6):3455–63. doi: 10.1245/s10434-023-13112-3 (PMC10175412; doi:10.1245/s10434-023-13112-3)
Supplement: Supplementary file 1 — Supplementary file1 (DOCX 58 kb) [file 10434_2023_13112_MOESM1_ESM.docx]

**SUPPLEMENTARY**

**FIG. S1** Case Report Form

**Table S1.** Change in resectability status after IOUS, according to the Dutch Pancreatic Cancer Group criteria


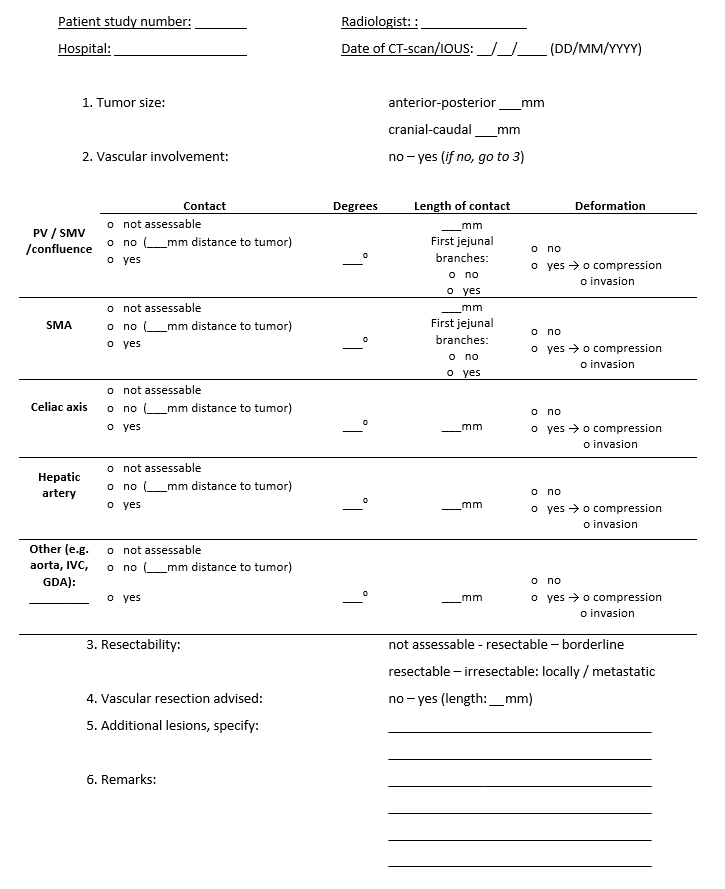
**Figure S1.** Case Report Form

*IOUS*: intraoperative ultrasound; *PV*: portal vein; *SMV*: superior mesenteric vein; *IVC*: inferior vena cava; *GDA*: gastroduodenal artery.

**Table S1.** *Change in resectability status after IOUS, according to the Dutch Pancreatic Cancer Group criteria*

|  |  | **Resectability status based on IOUS** | | |  |
| --- | --- | --- | --- | --- | --- |
|  |  | **Resectable** | **Borderline resectable** | **Locally advanced** | Total |
| **Resectability status based on preoperative imaging** | **Resectable** | 14 (88%) | 2 (12) | 0 (0%) | 16 (100%) |
|  | **Borderline resectable** | 26 (70%) | 11 (30%) | 0 (0%) | 37 (100%) |
|  | **Locally advanced** | 8 (25%) | 9 (28%) | 15 (47%) | 32 (100%) |
|  | Total | 48 (56%) | 22 (26%) | 15 (18%) | 85 (100%) |

The blue marked cells indicate the patients in which resectability status was downstaged after IOUS. The orange marked cells indicate the patients in which resectability status was upstaged after IOUS.

Overall change in resectability status: 45/85=53% (*p*<0.001).

*IOUS*: intraoperative ultrasound.
